# Identifying multi-locus chromatin contacts in human cells using tethered multiple 3C

**DOI:** 10.1186/s12864-015-1236-7

**Published:** 2015-02-25

**Authors:** Ferhat Ay, Thanh H Vu, Michael J Zeitz, Nelle Varoquaux, Jan E Carette, Jean-Philippe Vert, Andrew R Hoffman, William S Noble

**Affiliations:** Department of Genome Sciences, University of Washington, Seattle, 98195 WA USA; Veterans Affairs Palo Alto Health Care System, Stanford University Medical School, Palo Alto, 94304 CA USA; Mines ParisTech, PSL-Research University, CBIO-Centre for Computational Biology, 35 rue St Honoré, Fontainebleau, 77300 France; Institut Curie, Paris, F-75248 France; U900, INSERM, ParisF-75248, France; Department of Microbiology and Immunology, Stanford University, Stanford, 94305 CA USA; Department of Computer Science and Engineering, University of Washington, Seattle, 98195 WA USA

**Keywords:** Genome architecture, Chromatin conformation capture, Multi-locus chromatin contacts, Near-haploid human cells, Leukemia, Three-dimensional modeling

## Abstract

**Background:**

Several recently developed experimental methods, each an extension of the chromatin conformation capture (3C) assay, have enabled the genome-wide profiling of chromatin contacts between pairs of genomic loci in 3D. Especially in complex eukaryotes, data generated by these methods, coupled with other genome-wide datasets, demonstrated that non-random chromatin folding correlates strongly with cellular processes such as gene expression and DNA replication.

**Results:**

We describe a genome architecture assay, tethered multiple 3C (TM3C), that maps genome-wide chromatin contacts via a simple protocol of restriction enzyme digestion and religation of fragments upon agarose gel beads followed by paired-end sequencing. In addition to identifying contacts between pairs of loci, TM3C enables identification of contacts among more than two loci simultaneously. We use TM3C to assay the genome architectures of two human cell lines: KBM7, a near-haploid chronic leukemia cell line, and NHEK, a normal diploid human epidermal keratinocyte cell line. We confirm that the contact frequency maps produced by TM3C exhibit features characteristic of existing genome architecture datasets, including the expected scaling of contact probabilities with genomic distance, megabase scale chromosomal compartments and sub-megabase scale topological domains. We also confirm that TM3C captures several known cell type-specific contacts, ploidy shifts and translocations, such as Philadelphia chromosome formation (Ph+) in KBM7. We confirm a subset of the triple contacts involving the *IGF2-H19* imprinting control region (ICR) using PCR analysis for KBM7 cells. Our genome-wide analysis of pairwise and triple contacts demonstrates their preference for linking open chromatin regions to each other and for linking regions with higher numbers of DNase hypersensitive sites (DHSs) to each other. For near-haploid KBM7 cells, we infer whole genome 3D models that exhibit clustering of small chromosomes with each other and large chromosomes with each other, consistent with previous studies of the genome architectures of other human cell lines.

**Conclusion:**

TM3C is a simple protocol for ascertaining genome architecture and can be used to identify simultaneous contacts among three or four loci. Application of TM3C to a near-haploid human cell line revealed large-scale features of chromosomal organization and multi-way chromatin contacts that preferentially link regions of open chromatin.

**Electronic supplementary material:**

The online version of this article (doi:10.1186/s12864-015-1236-7) contains supplementary material, which is available to authorized users.

## Background

A variety of microscopic imaging techniques have long been used to study chromatin architecture and nuclear organization [1-3]. Recent advances triggered by the invention of chromatin conformation capture (3C) enable ascertainment of genome architecture on a genome-wide scale for virtually any genome, including human [4-6], mouse [5,7], budding yeast [8], bacteria [9], fruit fly [10] and a malarial parasite [11]. These studies have revealed that the three-dimensional form of the genome *in vivo* is highly related to genome function through processes such as gene expression and replication timing. Therefore, understanding how chromosomes fold and fit within nuclei and how this folding relates to function and fitness is crucial in gathering a thorough picture of epigenetic control of gene regulation for eukaryotic organisms.

Hi-C was the first molecular assay to measure genome architecture on a genome-wide scale [4], and the assay continues to be widely used [6,11,12]. Hi-C involves seven steps: (1) crosslinking cells with formaldehyde, (2) digesting the DNA with a six-cutter restriction enzyme, (3) filling overhangs with biotinylated residues, (4) ligating the fragments, (5) creating a sequence library using streptavidin pull-down, (6) high-throughput paired-end sequencing, and (7) mapping paired ends independently to the genome to infer contacts. A subsequently described assay by Duan et al. [8] is more complex, involving a pair of restriction enzymes (REs) applied in three separate steps (RE1, RE2, circularization, then RE1 again), as well as the introduction of EcoP151 restriction sites to produce paired tags of 25–27bp. More recently, the tethered conformation capture (TCC) assay enhances the signal-to-noise ratio by carrying out a Hi-C-like protocol using DNA that is tethered to a solid substrate [13].

One limitation of current genome architecture assays is their inability to identify simultaneous interactions among multiple loci. Chromosomes are composed of complex higher order chromatin structures that bring many distal loci into close proximity. In particular, evidence suggests that eukaryotic transcription occurs in factories containing many genes [14]. Recently, multiple-gene interaction complexes associated with promoters were found to contain an average of nearly nine genes [15]. However, currently available experimental data cannot ascertain to what extent these multiple gene interactions occur simultaneously or are confined to different sub-populations of nuclei. This distinction is analogous to the distinction between “party hubs” and “date hubs” in protein-protein interaction networks, in which a hub protein interacts either simultaneously or in a serial fashion with a series of partner proteins [16]. In the context of genome architecture assays, distinguishing between “party loci” and “date loci” will be a crucial first step in elucidating the role of combinatorial regulation of gene expression.

A molecular colony technique recently developed by Gavrilov et al. [17] investigated multicomponent interactions among remote enhancers and active *β*-globin genes in mouse erythroid cells. This assay, however, is PCR-based and requires a primer design step, which prevents it from providing a genome-wide picture of potential multicomponent contacts. An earlier genome-wide assay by Sexton et al., which is adapted from the traditional Hi-C protocol and is similar to the assay we present here, acknowledged the existence of multi-locus contacts that can be identified from paired-end reads in their data [10]. However, due to a number of differences in that protocol compared to TM3C (e.g., size selection for larger fragments, shorter read lengths and no in-gel ligation step), identifying a substantial number of multi-locus contacts was not possible when we apply our two-phase mapping pipeline to the Sexton et al. data (<0.0004% triples and no quadruples). Therefore, genome-wide methods that distinguish between simultaneous contacts among multiple loci and pairwise contacts that happen in different sub-populations of cells are still necessary.

**Figure 1:**
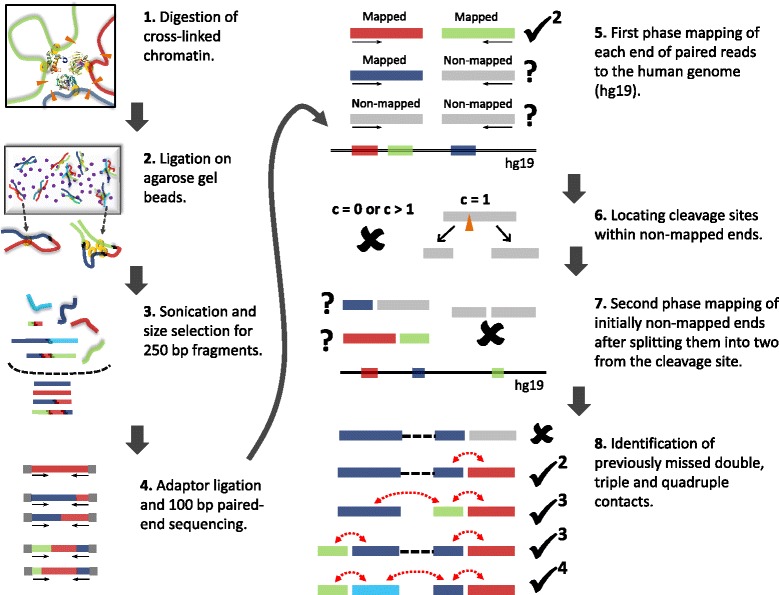
**Overview of TM3C experimental protocol and mapping of paired-end reads to human genome.** **1**. Cells are treated with formaldehyde, covalently crosslinking proteins to one another and to the DNA. The DNA is then digested with either a single 4-cutter enzyme (DpnII) or a cocktail of enzymes (AluI, DpnII, MspI, and NlaIII). **2**. Melted low-melting agarose solution is added to the digested nuclei to tether the DNA to agarose beads. Thin strings of the hot nuclei plus agarose solution is then transferred to an ice-cold ligation cocktail overnight. **3**. After reversal of formaldehyde crosslinks and purification via gel extraction, the TM3C molecules are sonicated and size-selected for 250 bp fragments. **4**. Size-selected fragments are paired-end sequenced (100 bp per end) after addition of sequencing adaptors. **5**. Each end of paired-end reads are mapped to human reference genome. If both ends are mapped then the pair is considered a *double* and retained because it is informative for genome architecture. **6**. Read ends that do not map to the reference genome are identified and segregated according to the number of cleavage sites they contain for the restriction enzyme(s) used for digestion. **7**. Reads with exactly one cleavage site are considered for the second phase of mapping. These reads are split into two from the cleavage site and each of these two pieces are mapped back to the reference genome. **8**. Read pairs with either one or both ends not mapped in the first mapping phase are reconsidered after second phase. Depending on how many pieces stemming from the original reads are mapped in the second phase, such pairs lead to either no informative contacts, *doubles*, *triples* or *quadruples*.

To address this issue, we developed the tethered multiple chromosome conformation capture assay (TM3C), which involves a simple protocol of restriction enzyme digestion and religation of fragments within agarose gel beads (tethering step) followed by high throughput paired-end sequencing (Figure 1, steps 1–4). We apply TM3C to two human cell lines and confirm that the DNA–DNA contact matrices produced by TM3C exhibit features characteristic of existing genome architecture datasets, including the expected scaling of contact probabilities with genomic distance, enrichment of intrachromosomal contacts, megabase scale chromosomal compartments and sub-megabase scale topological domains. We confirm that TM3C in KBM7 cells captures several known cell type-specific contacts, ploidy shifts and translocations, such as Ph+ formation. In addition, we demonstrate that TM3C enables genome-wide identification of contacts among more than two loci simultaneously. We identify multi-locus contacts involving three (triple) or four (quadruple) loci by a two-phase mapping strategy that separately maps chimeric subsequences within a single read (Figure 1, steps 5–8). This mapping strategy potentially allows us to identify co-regulation or combinatorial regulation events, while also greatly increasing the number of distinct pairwise contacts (doubles) identified. We also validate a subset of the triple contacts involving the *IGF2-H19* imprinting control region (ICR) using PCR for KBM7 cells. We demonstrate that pairwise and triple contacts prefer to link open chromatin regions to each other and regions with higher numbers of DHSs to each other.

Finally, we use the contact maps gathered from TM3C to infer a local 3D structure of the *IGF2-H19* region at 40 kb resolution and a whole genome 3D model at 1 Mb resolution for the near-haploid KBM7 genome. Our 3D models place *H19* and *IGF2* genes far away from each other, consistent with their opposite transcriptional status, and place gene-rich small chromosomes (chrs. 16, 17, 19–22) and large chromosomes (chrs. 1–5) near each other, confirming previous observations of gene-density- correlated arrangements of higher-order chromatin in human cells [18].

## Results

### Tethered multiple chromatin conformation capture (TM3C)

To identify simultaneous chromatin contacts among two or more loci, we digest crosslinked chromatin with one or more 4-cutter restriction enzymes (REs) (Step 1 of Figure 1). When using multiple REs, we select a set of enzymes such that sticky or blunt ends left by one enzyme are incompatible with the ends left by any other, thereby preventing ligation between fragments generated by different enzymes. We then encapsulate and ligate the digested DNA within agarose beads (Step 2 of Figure 1), which replaces the tethering step of Kalhor et al. [13]. We then size-select DNA fragments of around 250 bp and subject the selected fragments to high throughput paired-end sequencing (Steps 3, 4 of Figure 1). Our assay differs from the original Hi-C assay in three primary ways: (i) TM3C can use multiple REs simultaneously, (ii) TM3C does not include a step where sticky ends of restriction fragments are biotinylated, and (iii) TM3C carries out the ligation step within agarose gel beads. Digestion using multiple REs greatly increases the resolution that can be achieved via these genome-wide 3C-based techniques (Additional file 1: Figure S1). However, comparison of two libraries, one generated with four 4-cutters and the other with only one, suggests that the noise-to-signal ratio is much higher for the multiple 4-cutters case. Our second modification, elimination of the biotinylation step, greatly reduces the complexity of the overall protocol and has already been applied successfully by Sexton et al. [10]. This simplification, however, comes with the drawback of sequencing many uninformative, unligated sonication products both for the TM3C and the Sexton et al. protocols. Because detection of such uninformative read pairs is computationally trivial, this simplification, fortunately, does not contribute an additional noise factor. The third modification we implement, in-gel ligation, is similar to but simpler than the tethering achieved using protein biotinylation in the tethered conformation capture (TCC) assay [13]. Our initial experimental data which omitted the in-gel ligation demonstrated that without this step the resulting signal-to-noise ratio for the case of four 4-cutters is very low (95% of the contacts are interchromosomal). Addition of in-gel ligation step improved the percentage of intrachromosomal contacts from 5% to 20% and 48% for the four 4-cutter (KBM7-TM3C-4) and one 4-cutter (KBM7-TM3C-1) libraries, respectively. Therefore, we only present the results from the libraries generated using the in-gel ligation and focus mainly on the results from our one 4-cutter library for both KBM7 and NHEK cell lines.

We use TM3C to investigate the chromatin architecture of the near haploid cell line KBM7 (25, XY, +8, Ph+) extracted from a heterogeneous chronic leukemia cell line [19], and NHEK, a normal diploid human keratinocyte primary cell line (Lonza Walkersville Inc.). We construct libraries using only one four-base cutter restriction enzyme (TM3C-1) for both KBM7 and NHEK. We also create two libraries from KBM7 cells using four different four-base cutters, one from crosslinked cells (KBM7-TM3C-4) and one from non-crosslinked cells (KBM7-MCcont-4) as a control (Table 1). In what follows, we report results from application of TM3C to these two human cell lines mainly focusing on KBM7.

**Table 1:** **Summary of datasets generated in this paper**

**Cell type**	**Tethering**	**Restriction enzymes (REs)**				**Identifier**
		**AluI**	**MboI/DpnII**	**MspI**	**NlaIII**	
		**AG |CT**	**|GATC**	**C |CGG**	**CATG |**	
NHEK	Yes		*✓*			NHEK-TM3C-1
KBM7	Yes		*✓*			KBM7-TM3C-1
KBM7	Yes	*✓*	*✓*	*✓*	*✓*	KBM7-TM3C-4
KBM7 (gDNA)	No	*✓*	*✓*	*✓*	*✓*	KBM7-MCcont-4

### TM3C reveals multi-locus chromatin contacts

**Figure 2:**
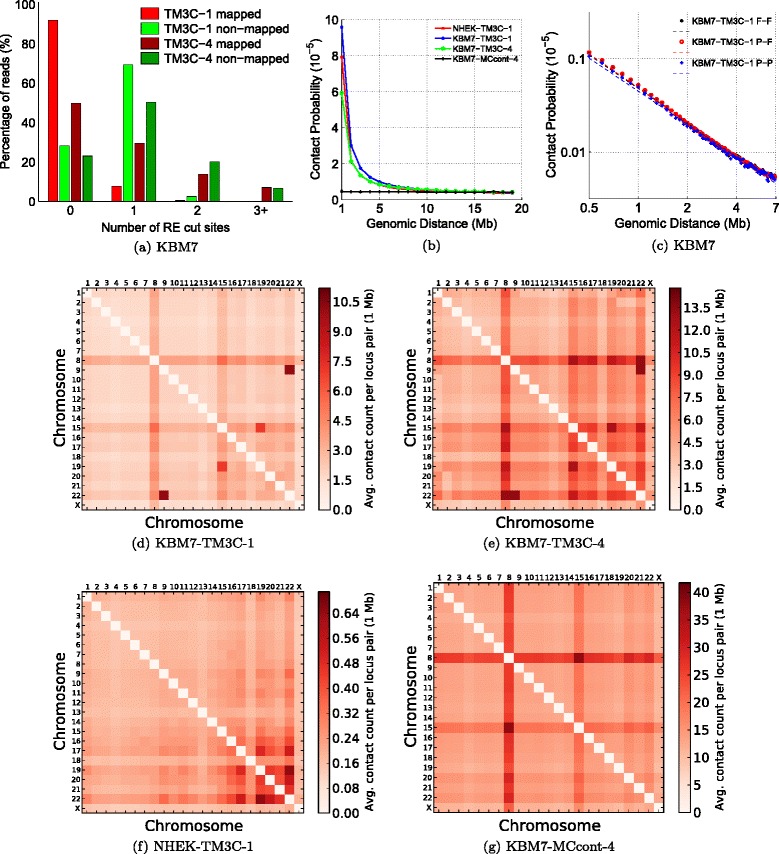
**Consistency of TM3C data with known organizational principles and KBM7 karyotype.** **(a)** Number of RE cut sites within reads that are fully mapped and nonmapped in the first phase mapping for KBM7 libraries. **(b)** Scaling of contact probability with genomic distance for three crosslinked libraries and one non-crosslinked control library. **(c)** Scaling of contact probability in log–log scale for three different sets of contacts identified in KBM7-TM3C-1 library. Pairwise chromosome contact matrices for **(d)** KBM7-TM3C-1, **(e)** KBM7-TM3C-4, **(f)** NHEK-TM3C-1 and **(g)** KBM7-MCcont-4 libraries. For these plots contact counts are averaged over all pairs of mappable 1 Mb windows between the two chromosomes.

In addition to providing higher resolution, the use of frequently cutting REs (4-cutters) or multiple REs together allows identification of simultaneous contacts among more than two loci, even with reads as short as 100 bp. The original Hi-C method only retains read pairs in which both reads map completely to the reference genome. Here we refer to this type of contacts as type **F-F** (fully mapped/fully mapped, Step 5 of Figure 1). Unlike current Hi-C mapping pipelines, after identifying **F-F** pairs, we further process the unmapped paired-end reads to see whether we can still rescue some informative chromatin contacts from them. Our motivation to pursue these reads stems from the striking difference between the number of restriction sites within fully-mapped versus non-mapped reads (Figure 2a). In both the TM3C-1 and TM3C-4 libraries, greater than 70% of the non-mapped reads contain at least one RE cut site, whereas 90% of the mapped reads contain no cut sites for the TM3C-1 library (two sample Kolmogorov-Smirnov test p-values for both TM3C-1 and TM3C-4 are approximately equal to 0). This difference suggests that read ends that fail to map as a whole can still be informative of chromatin contacts because they potentially contain real ligation events leading to chimeric reads. In order to extract this contact information, we further process the read ends containing one restriction site, thereby identifying contacts between a partially mapped read and a fully mapped read (**P-F**) or between two partially mapped reads (**P-P**, Steps 6–8 of Figure 1, Methods). This two-phase mapping strategy not only identifies a greater number of pairwise contacts (doubles) but also allows us to identify contacts involving three or four loci from only one paired-end read. Step 8 of Figure 1 summarizes the different cases arising from the second mapping phase for a read pair that did not qualify as F-F in the first phase. Overall, after excluding intrachromosomal contacts with genomic distance <20 kb, we identify more than 210K triples from our KBM7-TM3C-1 library together with 10.1M and 857K additional pairwise contacts from P-F and P-P type read pairs, respectively (Table 2, Additional file 2). We also investigate the mapping orientations (signs) of ligated fragments that create different contact types (Table 3). The distribution of reads among all possible sign combinations is expected to have a bias for reads that are sonication products (undigested or religated) and to be uniform for de novo chromatin contacts due to ligation events. Table 3 shows this is the case for both the contacts that are identified by traditional Hi-C pipelines (F-F) as well as for the contacts we identify here that produce triples. Since we size select for fragments that are approximately 250 bp, the genomic distance threshold of 1 kb eliminates all sonication products, resulting in uniform distribution for the remaining contacts from TM3C.

**Table 2:** **Summary of informative pairwise and multi-locus contacts for each KBM7 library**

**Library**	**Total reads**	**Doubles (pairwise)**	**Triples**	**Quadruples**
KBM7-TM3C-1	95,000,000	14,830,477	211,249	1,676
		(15.61%)	(0.22%)	(0.002%)
		inter: 8,036,033	inter: 92,959	inter: 672
		intra: 6,794,444	intra: 28,930	intra: 38
			mixed: 89,360	mixed: 966
KBM7-TM3C-4	72,800,218	13,858,985	816,625	25,158
		(19.04%)	(1.12%)	(0.034%)
		inter: 11,544,137	inter: 594,052	inter: 15,889
		intra: 2,314,848	intra: 22,787	intra: 85
			mixed: 199,786	mixed: 9,184

**Table 3:** **Summary of intrachromosomal read orientations for different contact types (KBM7-TM3C-1)**

**Contact type**	**Genomic dist.**	**Read orientations (end1/end2)**			
Doubles (F-F)		**+/+**	**+/-**	**-/+**	**-/-**
	All	1.8%	48.2%	48.2%	1.8%
	> 1 kb	24.9%	25.1%	25.1%	24.9%
Triples (F-P)		+/++,-/–	**+/+-,-/-+**	**+/-+,-/+-**	**+/–,-/++**
	All	0.1%	49.7%	0.2%	50%
	> 1 kb	24.5%	25.8%	25.3%	24.4%
Triples (P-F)		**++/+,–/-**	**++/-,–/+**	**+-/+,-+/-**	**+-/-,-+/+**
	All	0.2%	49.9%	49.7%	0.2%
	> 1 kb	25.6%	24.1%	25.4%	25.0%

### Two-phase mapping rescues contacts informative of genome architecture

Following identification of all three types of contacts (F-F, P-F, and P-P), we evaluate the quality of the resulting contact sets for each library in four ways. First, we confirm that the contact probability between two intrachromosomal loci exhibits a sharp decay with increasing genomic distance for crosslinked libraries but not for the control library when all contact types are pooled (Figure 2b). Second, we observe that this scaling relationship is consistent for different contact types (Figure 2c), and the scaling is log-linear for the genomic distance range of 0.5–7 Mb, consistent with observations from Hi-C data [4]. Third, we confirm visually and quantitatively that the interchromosomal contact maps we obtain from each contact type are consistent with each other (Additional file 1: Figure S2, pairwise matrix correlations are 0.997, 0.964 and 0.954 for (F-F, P-F), (F-F, P-P) and (P-F, P-P), respectively) and that the contact maps are consistent with known organizational hallmarks of human genome architecture, such as the increased number of contacts between small chromosomes (16–22 except 18) (Figure 2d–f, Additional file 1: Figure S2). Fourth, we confirm that our contact profiles capture known karyotypic abnormalities of KBM7 cells, such as diploidy of chromosome 8 (+8), partial diploidy of chromosome 15, and t(9;22)(q34;q11)) translocation between chromosomes 9 and 22 that leads to Philadelphia chromosome formation [19,20] (Figure 2d, e, Additional file 1: Figure S3). Normal diploid human keratinocyte (NHEK) cells exhibit no karyotypic abnormalities except higher average contact counts between chromosomes 17, 19 and 22 (Figure 2f). For the non-crosslinked KBM7 control library, only the changes related to copy number (e.g., diploidy) are apparent from the heatmap (Figure 2g). Translocations are not visible in the control because digestion of non-crosslinked chromatin does not preserve genomic distances. Together, these results indicate that TM3C successfully assays genome architecture of human cells and suggests that contacts recovered by our two-phase mapping strategy, which are traditionally discarded from Hi-C analysis, are consistent with traditionally retained contacts. Therefore, for all remaining analyses with pairwise contacts we combine all three types (F-F, P-F, P-P) into an aggregated contact map for each library.

### TM3C data confirms chromatin compartments and topological domains

**Figure 3:**
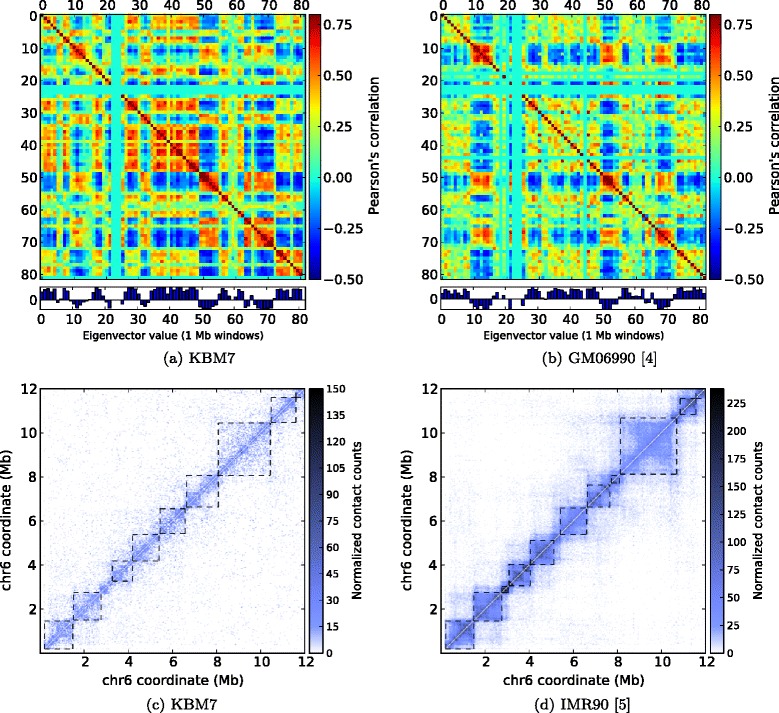
**Comparison of TM3C data with existing genome architecture datasets.** Eigenvalue decomposition to identify open/closed chromatin compartments of chromosome 17 **(a)** from the KBM7 cell line assayed by TM3C and **(b)** from GM06990 cell line assayed by Hi-C [4]. Topological domain calls and contact count heatmaps of a 6 Mb region of chromosome 6 **(c)** for the KBM7 cell line assayed by TM3C and **(d)** for the IMR90 cell line assayed by Hi-C [5].

In addition to evaluating whether results from the TM3C data sets are consistent with polymer models of chromatin folding and karyotypic properties of assayed cell lines, we assess whether TM3C contact maps exhibit the expected compartment-scale and domain-scale organization. For this purpose we perform eigenvalue decomposition on our contact maps and compare our compartment calls to those of previous Hi-C data sets on other human cell lines [4,5]. The resulting compartment calls exhibit a nearly perfect overlap for chromosome 17 between KBM7 and GM06990 (Figures 3a–b) and a high level of genome-wide conservation (82 *%*) between these two cell lines. Conservation between pairs of contact maps from the five previously published contact maps ranged between 70–82%.

Similarly, we perform topological domain decomposition at 40 kb resolution on KBM7 contact maps and compare our calls to those of two human cell lines published by Dixon et al. [5] (Methods). Figures 3c–d demonstrate the significant overlap of topological domain calls from KBM7 and IMR90 contact maps on a 12 Mb region of chromosome 6. Overall, 73% of IMR90 and 72.8% of ESC domain boundaries overlap with the boundaries that we identify for the KBM7 cell line (Fisher’s exact test p-values compared to random overlap are <10^−100^ for each case).

Together, the compartment-scale and domain-scale similarities between our data and previous Hi-C data suggests that TM3C, a simpler protocol, provides similar results to Hi-C and that KBM7, which has a distinct karyotype, preserves the large scale organizational features of other human cell lines.

### Genome-wide characterization of triple contacts

**Figure 4:**
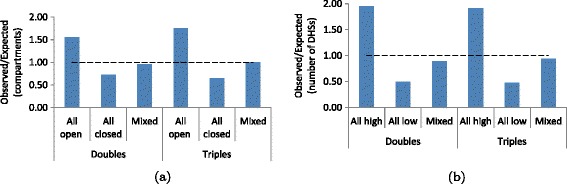
**Genome-wide characterization of triple contacts.** **(a)** Observed over expected percentages of double and triple contacts that link 1 Mb regions with the same (either open or closed) or different (mixed) compartment labels for the KBM7-TM3C-1 library (Methods). Both double and triple contacts prefer to link open compartments to each other with triples showing slightly more enrichment for this trend. **(b)** Similar percentages as in **(a)** but when 1 Mb windows are segregated according to the number of DHSs they contain (Methods). Contacts linking regions with higher numbers of DHSs than the median number are enriched within the doubles and the triples of the KBM7-TM3C-1 library. Due to lack of DNase data for KBM7 cells, we use data from six other human cell lines for this analysis. Since the results are very similar among different cell lines, here we only plot the results for K562 which is also a leukemia cell line.

After identifying chromatin compartments at 1 Mb resolution and topological domains at 40 kb resolution for the KBM7 cell line, we evaluate whether the triple contacts identified by TM3C preferentially link regions with the same compartment labels and regions within the boundaries of a topological domain. Figure 4a shows that triple contacts, similar to doubles, are enriched among regions of open chromatin (observed 14.6% compared to expected 8.33%, Methods). Out of all intrachromosomal triples (triples that link three loci on the same chromosome), we see that 16.5% are within the same topological domain. Note that we exclude from this percentage all short range intrachromosomal triples (<20 kb) as well as all those that link at least two loci within the same 40 kb window which would otherwise inflate the reported percentage. We assess the significance of this observed percentage of intradomain triples by generating a null model with 100 shuffled topological domain decompositions for each chromosome (Methods). The median and the mean percentages are both ∼14.1% with a standard deviation of 0.16% for the null model suggesting a statistically significant enrichment of intradomain triples for the observed domain decomposition compared to shuffled configurations (p-value =0, z-score =14.67).

Next we carry out an analysis similar to the compartment label analysis described above using the numbers of DNase hypersensitive sites within each 1 Mb window (Methods). Figure 4b shows that, consistent with and slightly surpassing the enrichment for open chromatin compartments, triple contacts as well as doubles are enriched among regions with higher numbers of DHSs (for triples observed 23.7% compared to expected 12.4%, Methods).

### Verification of triples involving *IGF2-H19* locus

**Figure 5:**
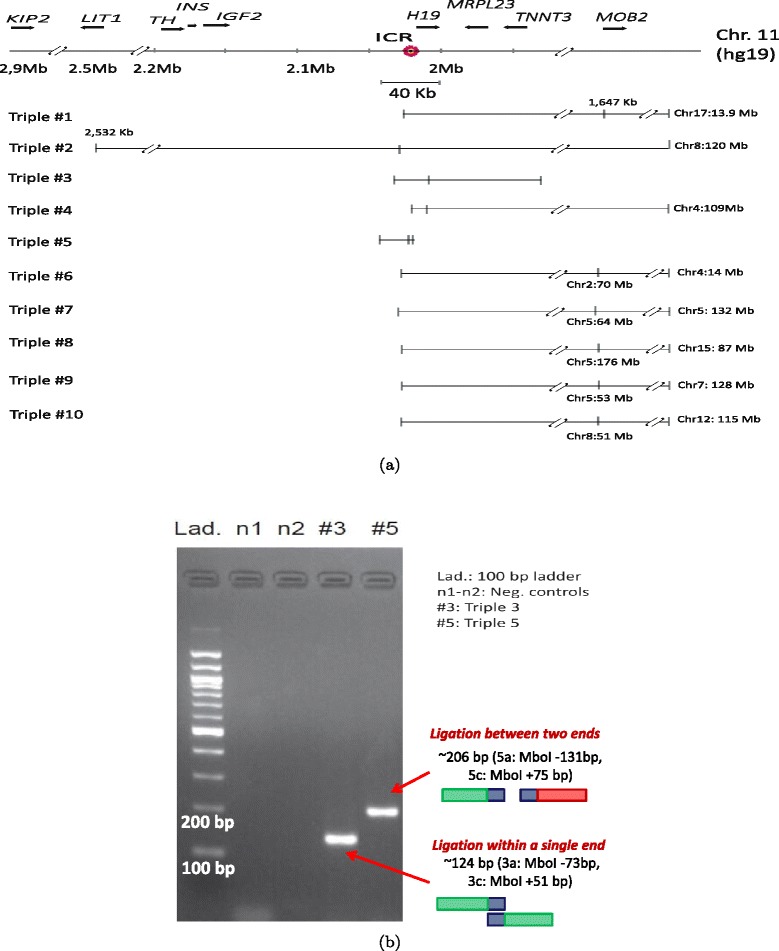
**Validation of triples using PCR.** **(a)** Ten triples extracted from the KBM7-TM3C-1 library that have at least one of their three ends in the 40 kb region surrounding the imprinting control region (ICR) of *IGF2* and *H19* genes. These triples involve short- and long-range contacts within chromosome 11 which are all indicated by tick marks with coordinates in kilobases (kb) displayed only for long-range contacts. Interchromosomal contacts with other chromosomes are indicated by the chromosome identifier followed by the coordinate in megabases (Mb). Orientation of the displayed locus is in the direction of *IGF2* and *H19* transcription. **(b)** PCR verification of pairwise contacts from triples 3 and 5. One pair of forward/reverse primers is used for each gel (Additional file 1: Table S1).

We next investigate whether the multi-locus contacts identified by the TM3C assay correspond to possible combinatorial regulatory interactions in KBM7 cells. Specifically, we focus on triples (contacts involving three loci) involving the *IGF2-H19* locus, which is a classic example of imprinting that leads to allele-specific gene expression and regulation in both mouse and human [21-24]. Our previous work in human cells has shown that a region that is located just upstream of the *H19* promoter which is differentially methylated between maternal and paternal copies is involved in formation of allele-specific long-range chromatin loops [23]. Methylation status of this imprinting control region (ICR) determines whether *IGF2* is transcribed (paternal allele) or not (maternal allele). Because KBM7 cells are haploid for chromosome 11, we expect our TM3C data to be consistent with only one mode of operation of this ICR. Analyzing the triples inferred from KBM7-TM3C-1 data involving the ICR region (±20 kb), we observe contacts that link this ICR region to distal loci on the same chromosome as well as to a *trans* loci on other chromosomes (Figure 5a).

In order to verify these contacts, we design three primers per each triple and perform PCR experiments (Additional file 1: Table S1). We test whether pairs of forward/reverse primers give rise to PCR products with expected sizes to confirm contacts identified from our two-phase mapping (Figure 5b). For triple 3, we use primers 3a and 3c designed for two loci that are 80 kb away and are linked by a contact found from a ligation occurring within one end of a paired-end read. For triple 5, we use primers 5a and 5c that link two loci that are 24 kb apart on chromosome 11 and are found (one of them only partially) in two separate ends of a paired-end read. For both of these cases we observe PCR products near the expected size from our primer design (Additional file 1: Table S1). Validation of contacts found by our two-phase mapping either within a single end of a read or from two different ends supports the idea that chimeric reads contain information about genuine chromatin contacts.

**Figure 6:**
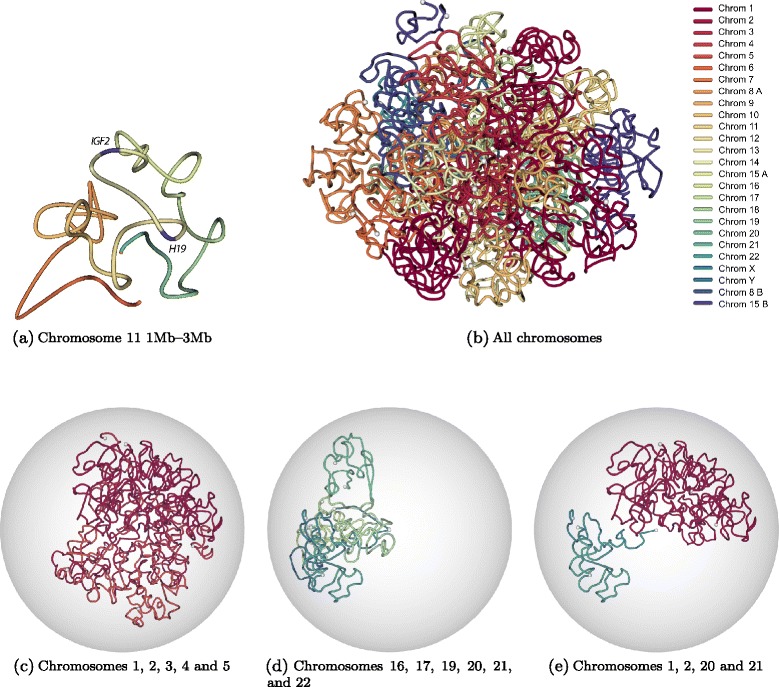
**Three-dimensional modeling of KBM7 genome architecture.** **(a)** Three-dimensional structure of the 2 Mb region of chromosome 11 (chr11:1,000,000-3,000,000) which is centered around *IGF2-H19* imprinting control region. This structure is inferred from normalized contact counts of KBM7-TM3C-1 data at 40 kb resolution using the Poisson model from Varoquaux et al. [27]. **(b)** Three-dimensional structure of the KBM7 genome, which is haploid for all chromosomes other than diploid chromosome 8 (8A, 8B) and partially diploid chromosome 15 (15A, 15B) (see Methods for details of the 3D inference). Different colors represent different chromosomes, and white balls represent chromosome ends. Same 3D structure as (b) when confined to **(c)** only a subset of long chromosomes, **(d)** only a subset of small chromosomes, **(e)** two small and two large chromosomes.

Next, we perform PCR on all the triples shown in Figure 5a using all three primers simultaneously. Out of 10 triples tested, 6 of them (triples 1–6) resulted in either one or more PCR products that have the expected size(s), confirming these contacts (Additional file 1: Figures S4, S5 and Table S1). Detailed analysis of the distal loci that are contact partners of ICR (either interchromosomal or interchromosomal with distance >40 kb to ICR) in these six triples reveal that most of these loci (6 out of 8, Additional file 1: Figures S6–S8) lie in regions consisting mainly of unmethylated CpGs in K562 cells and mainly methylated CpGs in at least one other cell line assayed by ENCODE [25]. These contacts suggest existence of complex chromatin loops that bring together the differentially methylated ICR in 3D with loci that show cell type-specific methylation and specifically unmethylation in K562 cells. These results together with our preliminary methylation analysis of the ICR suggest a 3D organization which silences *IGF2* by restricting enhancer access to its promoter similar to *Igf2* silencing of the maternal copy of mouse chromosome 7 [26]. In order to test our hypothesis that the single copy of chromosome 11 in KBM7 corresponds to the maternal allele, we check the expression status of *H19* and *IGF2* genes from a recently published data set [20]. Additional file 1: Figure S9 shows that *H19* is expressed but *IGF2* is not as we expected. This expression data confirms the prediction from TM3C data for the parent-of-origin of chromosome 11 in KBM7 cells. Furthermore, our 3D model of the 2 Mb region centered on the ICR (Figure 6a) demonstrate that the two genes, expressed *H19* and non-expressed *IGF2*, are placed in distal chromatin domains, consistent with the proposed gene regulation model in the maternal copy of the homologous region in mouse [24]. However, the depth of our data is not sufficient to do a finer scale 3D modeling that can distinguish between allele specific loops established by several differentially methylated regions and CTCF binding sites.

### Three-dimensional modeling of KBM7 genome recapitulates known organizational principles of human cells

Finally, to visualize the genome architecture of near-haploid KBM7 cells, we generated a set of 3D structures using an optimization framework that alternates between inferring the 3D configuration of beads that best summarize TM3C contacts [27] and re-estimating the distribution of contact counts between diploid chromosomes. Since this optimization is non-convex, we ran the optimization 1000 times and selected the 100 structures with the highest log likelihoods (Methods). Figure 6b–e plots the structure with highest likelihood inferred at 1 Mb resolution. Visual observation of Figure 6b suggests that individual chromosomes preserve their territories in 3D (see also Additional file 3). In order to better visualize which chromosomes are closer to each other, we plot subsets of different chromosomes in Figures 6c–e. Consistent with previous models [4] and our contact count heatmaps, we observe strong colocalization among the small gene-rich chromosomes (16, 17, 19, 20, 21 and 22). However, chromosome 18, which is small but gene-poor, does not colocalize with gene-rich small chromosomes in 3D (Figure 6c, Additional file 1: Figure S10). We also observe colocalization of large chromosomes with each other, but not as strongly as small chromosomes (Figure 6d). Visualization of two large and two small chromosomes clearly demonstrates that the two sets of chromosomes are far from each other in our 3D models (Figure 6e).

## Discussion

Catalyzed by the availability of genome-wide chromatin architecture data generated using chromatin conformation capture assays, the field of regulatory genomics has recently witnessed increased interest in the functional role of higher order DNA structure. Organizational principles of eukaryotic nuclei that are uncovered by these genome wide assays range from large scale patterns such as open/closed chromatin compartments [4] and topological domains [5] to more local patterns such as silencing or activating of individual genes by altering the 3D proximity of enhancers to gene promoters [15,28]. However, one important question that remains to be answered is how the simultaneous proximity of more than two loci in the nucleus impacts gene regulation. Current conformation capture assays cannot address this question because they only characterize pairwise contacts that involve exactly two loci.

Here we demonstrated how to discover simultaneous multi-locus contacts using a straightforward conformation capture assay. We aimed at distinguishing between proximity of multiple loci measured from different nuclei in the form of pairwise contacts and simultaneous proximity between these loci within a single nucleus. We showed that our TM3C assay, which can employ more than one restriction enzyme at a time to increase chromatin digestion, results in chimeras even within a single end of a short paired-end read. Accordingly, we developed a two-phase mapping pipeline that uses cleavage information to extract from these chimeras informative contacts that involve two, three or four loci. An additional advantage of TM3C is that it is significantly simpler and yet provides increased resolution for the resulting contact maps compared to current Hi-C assays.

It is important to note, however, that there are two drawbacks to our assay compared to traditional Hi-C or TCC assays. The first drawback is a tradeoff between resolution and the noise level of the data. Frequent digestion of chromatin with multiple 4-cutters increases the resolution but also the noise level of the data, as measured by the ratio between inter and intrachromosomal reads (Additional file 2). The second drawback is a tradeoff between the simplicity of the assay and the proportion of informative reads from the paired-end sequencing. In the TM3C assay we omit the steps of RE overhang biotinylation and streptavidin pull-down which are present in both the Hi-C and TCC assays. This omission results in a higher percentage of sonication products (non-informative read pairs) in the sequencing libraries of TM3C (Additional file 2) which we discard after read mapping.

Despite these drawbacks, we believe that TM3C is an effective assay in profiling genome architecture—evident by the consistency of our results with characteristic features of genome organization—with the added benefit of revealing multi-locus contacts. In order to demonstrate the utility of TM3C, we applied it to two human cell lines. We specifically chose one of these cell lines as the near-haploid KBM7 which has been used in settings where having multiple copies of a chromosome is problematic, such as loss-of-function genetic screens [20,29]. We first established that TM3C contact maps are consistent with karyotypic features of KBM7 and that KBM7 cells share common large scale organization with other mammalian cell lines previously assayed by Hi-C. Focusing on a well-studied locus (*IGF2-H19*) that has been shown to be involved in parent of origin specific long-range chromatin loops, we showed that TM3C identifies multi-locus contacts (triples), more than half of which were validated using PCR. Confirmed triples involved intrachromosomal loops bringing together regions that are more than megabases away in genomic distance as well as regions from different chromosomes. Together with results from previous FISH experiments that reveal *IGF2* is located outside of its chromosome territory in the majority of nuclei [30], our findings suggest that complex regulation of *IGF2* and *H19* may involve interactions with multiple distal regions simultaneously.

Another important aspect of our work is the modeling of 3D organization of a human cell line without averaging data from multiple copies of a chromosome or resolving the haplotype. To date 3D modeling efforts on the human genome have been limited to haploid chromosomes such as the X chromosome in male cells [31], one chromosome or one portion of a chromosome at a time [31,32] or have assumed artificially that only one copy of each chromosome exists per cell [33]. In this work the near-haploid karyotype of KBM7 allowed us to overcome these limitations to infer whole-genome 3D models. By extending an algorithm that we developed previously for haploid genomes [27] to handle the diploid portions of KBM7 cells, we generated 3D models for this leukemia cell line. Due to the lack of independent data available on KBM7 cells, we were unable to verify our 3D models further or correlate them with features such as histone modifications and transcription binding. However, our models are consistent at the large scale with previous observations that suggest chromosomes with similar sizes tend to be closer to each other in 3D. It is also important to note that, similar to many previous approaches, our 3D models are consensus structures that summarize the genome architecture of a cell population. Capturing the heterogeneity of genome architecture across cells may be possible in the future, especially in conjunction with single-cell techniques [31].

Overall, we showed that TM3C provides a framework to identify multi-locus contacts genome-wide in conjunction with commonly used next generation sequencing platforms that produce short paired-end reads (e.g., 100 bp Illumina). We believe that with broader use of longer reads (e.g., Pacific Biosciences) TM3C will be able to profile a larger number of multi-locus contacts with higher signal-to-noise ratio. Such profiling is important in understanding better the combinatorial regulation of gene expression and complex chromatin loops that involve more than two loci simultaneously.

## Conclusion

TM3C is a simple protocol for ascertaining genome architecture and can be used to identify simultaneous contacts among three or four loci. Application of TM3C to a near-haploid human cell line revealed large-scale features of chromosomal organization and multi-way chromatin contacts that preferentially link regions of open chromatin.

## Materials and methods

### TM3C library generation

Approximately six million NHEK and ten million KBM7 cells were fixed in 1.5% formaldehyde at room temperature for 10 minutes. The fixed cells were washed with TN buffer (10 mM Tris, 40 mM NaCl, pH 7.5) and collected by centrifugation at 600 g for 3 minutes. To increase digestion efficiency, fixed cells (6 or 10 million / 122 ul) were treated with SDS (add 3.8 ul of 10% SDS to a final of 0.30% SDS) at 64°C for 10 minutes and then at 37°C overnight (15 hours). The SDS concentration was reduced gradually to 0.10% by adding five times of 50 ul (1 x DpnII digestion buffer or NEB buffer 4 for multiple enzymes) with mixing. Triton X-100 (38 ul of 20% Triton X) was added to 1.8% concentration and the sample was incubated at 37°C for 1 hour. Sample volume was adjusted to 600 ul by adding 1 X restriction buffer, ATP (0.2 mM final) and BSA (100 ug/ml final). Digestion with appropriate restriction enzymes (300 units each) was carried out on a rotate shaker at 37°C for 15 hours. We used high concentration NEB enzymes to keep the final volume of the enzyme mixture less than 60 ul (1/10 reaction volume).

The digested samples were deactivated at 65°C for 15 minutes and then centrifuged at 15,000 g for 5 min. We recovered ∼95% of cellular DNA in the pellet fraction. The pellet fraction was re-suspended with T4 ligation buffer (15 ul 10 x buffer, 65 ul total) heated at 65°C and mixed with 100 ul of melted 2.5% low-melting agarose. We used 200 ul pipette to deliver the hot agarose sample to ice-cold ligation buffer (800 ul of 1 x ligation buffer containing T4 ligase (4000 units, NEB) in a steady fashion within ∼5 seconds, on melted ice. Strings of gel bead appeared instantly at 0°C. We sealed the tube with parafilm and perform ligation at RT (23°C.) overnight on top of a shaker (∼300 rpm), then transfer the tube to a iced water bath.

The sample pellet was recovered by centrifugation at 20,000 g for 2 minutes, then 10 ul of 1% SDS (0.05% final) was added and heated at 80°C for 1 hour. Cross-links were reversed by treatment with Proteinase K (200 ug/ml) at 65°C and 300 rpm overnight (12 hours). Melted TM3C-agarose sample was incubated with RNase A (10 ug / 210 ul) at 55°C for 15 minutes and then purified by QIAquick gel extraction protocol (QUIAGEN Inc., CA). Purified TM3C DNA was quantified using both a NanoDrop spectrophotometer (Thermo Scientific) and a Qubit 2.0 Fluorometer. The Qubit quantification represents the more accurate DNA concentration.

### First phase mapping of sequence data

We mapped the paired-end reads to the human reference genome (hg19) using the short read alignment mode of BWA (v0.5.9) with default parameter settings. Each end of the paired reads was mapped individually. We post-processed the alignment results to extract the reads that satisfy the following three criteria: (i) mapped uniquely to one location in the reference genome, (ii) mapped with an alignment quality score of at least 30, (iii) mapped with an edit distance of at most 3. Reads that satisfy these criteria are named *fully-mapped* (**F**), and the rest of the mapped reads that did not satisfy these criteria are discarded from further analysis. We identified pairs of fully-mapped reads that share a common identifier to generate the set of contacts that we denote as **F-F** (fully-mapped - fully-mapped). The reads that did not map to any location in this phase of mapping are named *non-mapped* and are analyzed further.

### Second phase mapping of non-mapped reads

Re-mapping the reads that are deemed *non-mapped* in the initial mapping is necessary to avoid discarding a significant number of informative reads for an assay such as TM3C that uses a frequently cutting restriction enzyme (or enzymes) for digestion. Due to the high frequency of cleavage sites in the genome, TM3C is highly likely to capture ligations between DNA fragments from two different loci in a single end of a read. We call each such read *chimeric* because the sequences do not come from a continuous piece of DNA but instead from two loci that are in proximity in the three-dimensional space. Therefore, for these chimeric ends, after splitting into smaller fragments from the cleavage sites of the restriction enzymes used in the digestion step, we applied a second phase of mapping.

Within each non-mapped read, we first counted the number of cleavage sites, taking into account all the restriction enzymes that are used in the digestion step for that specific library. We discarded reads that contain more than two cleavage sites. We also discarded reads that contain no cleavage sites because such reads surely are not chimeric. We split the remaining reads that contain only one cleavage site into two smaller fragments, preserving the entire cleavage site on both adjacent fragments. We mapped the two resulting fragments to the genome using BWA with default parameter settings. The 3-point filtering criteria mentioned in the previous section are applied to the aligned reads, but allowing an edit distance of at most 1 to make sure we only extract the unique and high quality mappings. The reads that are extracted from this phase of mapping are named *partially-mapped*(**P**) because they did not map as a whole, but their constituent fragments were successfully mapped to different loci. The two classes of mapped reads (fully-mapped (**F**) and partially-mapped (**P**)) yield three possible types of contacts, namely **F**-**F**, **F**-**P** and **P**-**P**. The first set (F-F) is extracted after the initial mapping in which each paired-end read can contribute at most one interaction between two loci. The second set (P-F) consists of paired-end reads with one end fully mapped and the other end having either one or two smaller fragments that mapped to the genome. If the latter contains only one mapped fragment, then the only interaction is between this fragment and the fully-mapped end. However, if the end has two mapped fragments, then this paired-end read produces three contacts: one between the two mapped fragments on the partially-mapped end and two others that have one side from a fragment from the partially-mapped end and the other side from the fully-mapped end. In addition, the same paired-end read produces one triple (i.e., interaction among three loci) of type P-F. For the contacts of the third type (P-P), each paired-end can produce either one, three or six pairwise contacts, depending on whether one or two fragments from each end are successfully mapped. If only one fragment from one end and two from the other is mapped, then, similar to the case of P-F, three pairwise contacts and one triple is produced. If both ends have two mapped fragments, then six pairwise contacts, four triples (of type P-P) and one quadruple (i.e., contact among four loci) are produced.

### Normalization of contact maps

For each possible pair of 1 Mb loci, we refer to the total number of read pairs that link the two loci as the *contact count*, and we refer to the two-dimensional matrix containing these contact counts as the *raw contact map*. To normalize the 3113×3113 raw contact maps, we extended the iterative correction procedure, ICE [34], for a nearly haploid genome. First, we corrected for the bias caused by the partial diploidy of the genome. For that, we constructed a “deduplicated” contact counts matrix, where contact counts associated with diploid loci are divided into two equal parts, each of which is associated with one of the homologous chromosomes. Contact counts between two different copies of diploid chromosomes/regions are set to 0. The deduplicated matrix is akin to an artificially created allele-specific contact counts matrix, where homologous chromosomes interact in identical ways and do not interact with each other. As a preprocessing step, we ranked loci by their percentage of intrachromosomal contacts with zero counts and filter out the top 10% of this list. This filtering removes all loci for which the signal to noise ratio is too low (typically, regions of low mappability). Last, we applied ICE, a method that attempts to eliminate systematic biases in Hi-C data. ICE assumes that the bias for each entry can be decomposed as the product of the biases associated with each locus, and estimates a bias vector *β* under the equal visibility hypothesis: the coverage of counts should be uniform. The tensor product *β*⊗*β* generates a bias matrix that can be used to convert the raw contact map into a normalized contact map. To generate a contact count matrix of the original size, we summed all counts from homologous chromosomes associated with the same loci. This procedure yields a (3113×3113) contact counts matrix for which diploid loci interact twice as much as haploid loci.

### Eigenvalue decomposition

We carried out eigenvalue decomposition on the normalized contact maps of KBM7 and NHEK TM3C datasets as described in [4]. For each chromosome we used the intrachromosomal contact matrices at 1 Mb resolution. We calculated the Pearson correlation between each pair of rows of the contact matrix and apply eigenvalue decomposition (using the eig function in MATLAB) to the correlation matrix. The sign of either the first or the second eigenvector defines chromosome compartments for each chromosome. Similar to [4], we used the second eigenvector in cases where the first eigenvector values are either all positive or all negative. To map signs of eigenvectors to open/closed compartment labels we used GC content as a marker. For each chromosome the sign with higher GC content is selected as open chromatin. We then compared the percentage of 1 Mb bins that are assigned the same compartment label by TM3C data versus previously published Hi-C data in four human cell lines (H1-hESC, IMR90 [5]; K562, GM06990 [4]).

### Topological domain analysis

We identified topological domains using a previously described hidden Markov model-based software tool [5]. To facilitate direct comparison with the previously published topological domains in human cell lines, we carried out the domain calling for these published datasets using the human GRCh36/hg19 assembly. We applied the topological domain calling on normalized contact maps of our TM3C data at 40 kb resolution. To measure the consistency between the topological domains inferred from TM3C and those from published Hi-C data, we calculated the overlap of domain boundaries obtained between these two assays. We deemed two boundaries, one from each assay, as overlapping if they overlap by at least 1 bp or are adjacent to each other, as described in [5].

### Contacts among regions with the same compartment label

We used compartment labels assigned by the eigenvalue decomposition as described above and computed the number of read pairs that define double and triple contacts between two or among three regions all with the same compartment label (all open or all closed) or at least two with opposite labels (mixed). We used only interchromosomal doubles and interchromosomal triples (linking three different chromosomes) for this analysis and eliminated regions that have less than 50% uniquely mappable bases. We then computed the number of all possible pairs and triples of 1 Mb windows and segregated this number into three groups (all open, all closed, mixed) giving us the expected percentages of contacts that should fall into each group. With exactly equal numbers of open and closed compartments for each chromosome, these percentages would be 25%, 25%, 50% for pairs of compartments and 12.5%, 12.5%, 75% for triples of compartments for the groups of all open, all closed and mixed, respectively. We then reported the ratio between the percentage of observed double and triple contacts to expected percentages within each of these three groups. A ratio >1 represents an enrichment for the observed contacts for that compartment label group.

### Contacts among regions with similar numbers of DHSs

We performed an analysis similar to the compartment label analysis described above using joint (UW–Duke) DNase hypersensitivity peak calls for the six Tier 1 cell lines (GM12878, H1-hESC, HeLa-S3, HepG2, HUVEC, K562) downloaded from http://ftp.ebi.ac.uk/pub/databases/ensembl/encode/integration_data_jan2011/byDataType/openchrom/jan2011/fdrPeaks. Since there is no DNase data for KBM7 we reported results for only K562 which is also a leukemia cell line. We computed for each 1 Mb window with mappability of at least 50% the number of DHS peaks that overlap with this window. We sorted all these windows by decreasing number of DHSs and labeled the top 50% as “high” and bottom 50% as “low” DNase sensitivity. We then calculated and reported the expected over observed percentage of doubles and triples as described for compartment labels.

### Contacts within the same topological domain

After carrying out the topological domain calling using our KBM7-TM3C-1 data, we computed the percentage of intrachromosomal doubles and triples that link loci within the same topological domain. To estimate the significance of the observed percentages, we randomly shuffled topological domains by preserving the distribution of the domain lengths for each chromosome arm as described in Ay et al. [35]. We reported the mean and the standard deviation for the percentage of within domain doubles and triples across 100 randomized shufflings.

### Inference of the 3D structure

We modeled each chromosome as a series of beads on a string, spaced approximately 1 Mb apart. We denote by $\textbf {X} = (x_{1},\ldots,x_{n}) \in \mathbb {R}^{3 \times n}$ the coordinate matrix of the structure, where *n* denotes the total number of beads in the genome including the newly introduced chromosomes 8B and 15B (*n*=3289 for the KBM7 genome), and $x_{i}\in \mathbb {R}^{3}$ represents the 3D coordinates of the *i*-th bead. Contacts from TM3C data can be summarized as an *m*×*m* matrix **c**, where each entry *c*_*kl*_ corresponds to the observed contact count between loci *k* and *l*. Because contact information does not distinguish between homologous chromosomes, *m* only includes one copy of each chromosome and *m*<*n*. For loci in diploid regions, the contact counts are the sum of contact counts due to each copy of the region. If we denote by *Φ*:[1,*n*]→[1,*m*] the mapping that associates a bead *i* to a locus *Φ*(*i*) of the contact count matrix, this means that the contact count *c*_*kl*_ between loci *k* and *l* is the sum of counts due to interactions between beads in *Φ*^−1^(*k*) and *Φ*^−1^(*l*). For any two beads *i* and *j* mapping respectively to loci *k*=*Φ*(*i*) and *l*=*Φ*(*j*), let us denote by 0≤*μ*_*ij*_≤1 the proportion of counts in *c*_*kl*_ due to interactions between beads *i* and *j*. Since all contact counts must be accounted for by interactions between beads, we must have for any loci *k* and *l*:

$$\sum\limits_{i\in\Phi^{-1}(k)\,,j\in\Phi^{-1}(l)} \mu_{ij}=1\,. $$

We propose to jointly infer the structure **X** and the distributions of contact counts *μ*_*ij*_’s by maximizing the likelihood of the observed contact counts. For that purpose, we modeled the contact frequencies $(\mu _{\textit {ij}}c_{\Phi (i) \Phi (j)})_{(i,j) \in \mathcal {D}}$ ( is the set of non-zero contact counts) as independent Poisson random variables, where the Poisson parameter of *μ*_*ij*_*c*_*Φ*(*i*)*Φ*(*j*)_ is a decreasing function of the Euclidean distance *d*_*ij*_(**X**) between beads *i* and *j*. Our and others’ previous work suggested that the relationship between *μ*_*ij*_*c*_*Φ*(*i*)*Φ*(*j*)_ and *d*_*ij*_ is approximately of the form *d*_*ij*_(**X**)^*α*^, with *α*=−3 [4,27,36]. We can then express the likelihood of the model as:

(1) $$ \ell(\mathbf{X}, \mu) = \prod\limits_{i, j} \frac{\left(d_{ij}^{\alpha}\right)^{\mu_{ij} c_{\Phi(i)\Phi(j)}}} {\left(\mu_{ij} c_{\Phi(i)\Phi(j)}\right)!} \exp \left(- d_{ij}^{\alpha}\right) \,.  $$

To infer the position of each bead, we maximized the log likelihood of the model which is:

(2) $${} {\fontsize{9pt}{9.6pt}\selectfont{\begin{aligned} \mathcal{L}(\mathbf{X}, \mu) = \sum\limits_{i, j} \mu_{ij} c_{\Phi(i)\Phi(j)} \alpha \log(d_{ij}) - d_{ij}^{\alpha} - \log\left(\mu_{ij} c_{\Phi(i)\Phi(j)}!\right)\!. \end{aligned}}}  $$

In practice, we solved the following relaxation since *μ*_*ij*_*c*_*Φ*(*i*)*Φ*(*j*)_ may not have integer values

(3) $$ \begin{aligned} \mathcal{L}(\mathbf{X}, \mu) =& \sum\limits_{i, j} \mu_{ij} c_{\Phi(i)\Phi(j)} \alpha \log\left(d_{ij}\right) - d_{ij}^{\alpha}\\ &- \log\left(\Gamma\left(\mu_{ij} c_{\Phi(i)\Phi(j)} + 1\right)\right) \,, \end{aligned}  $$

with the following constraints:

- *d*_*ij*_≤*d*_*max*_. To find a suitable *d*_*max*_, we first computed the expected distances $c_{i, i+1}^{-1 / 3}$ for adjacent beads of haploid chromosomes. We set *d*_*max*_ to the 97% quantile, thus excluding outliers values arising in the normalization procedure.
- 0.3≤*μ*_*ij*_≤0.7, where *i* and *j* corresponds to loci from the same copy of a diploid chromosomes.

To optimize this non-convex function, we iterated between two steps: (1) infer the 3D structure **X**; (2) re-estimate the distribution of contact counts *μ*_*ij*_ between diploid chromosomes. The first step is solved using an interior point method, as described in [27]. For the second step, the optimization problem can be performed with respect to each pair of loci *k* and *l* independently. Thus we perform a grid search on {*μ*_*ij*_|*Φ*(*i*)=*k*,*Φ*(*j*)=*l*}, with a step size of 0.01.

We ran the optimization 1000 times varying the initialization of the distribution of the contact counts, and another 1000 times varying the initial structure **X**. We then selected the top 100 structures with the highest log likelihoods.

## Additional files

Additional file 1
**Supplementary information.** This file contains supplementary figures and tables.

Additional file 2
**Summary of the two-phase mapping results.** This file contains separate worksheet describing in detail the numbers of reads processed at each step of our two-phase mapping.

Additional file 3
**Rotating view of our KBM7 3D model.** This file contains a movie of the KBM7 3D structure that resulted in the highest log likelihood inferred by our algorithm. Each chromosome is colored as indicated in Figure 6b.

## Abbreviations

3C: Chromatin conformation capture
TM3C: Tethered multiple 3C
TCC: Tethered 3C
ICR: Imprinting control region
PCR: Polymerase chain reaction
RE: Restriction enzyme
Ph+: philadelphia chromosome positive
CpG: Cytosine—phosphate—Guanine
DHS: DNase hypersensitive site
TSS: Transcription start site
