# Supplementary material for: Identifying multi-locus chromatin contacts in human cells using tethered multiple 3C
Source: BMC Genomics. 2015 Feb 25;16(1):121. doi: 10.1186/s12864-015-1236-7 (PMC4369351; doi:10.1186/s12864-015-1236-7)
Supplement: Additional file 1 — Supplementary information. This file contains supplementary figures and tables. [file 12864_2015_1236_MOESM1_ESM.pdf]

Supplementary Information

Identifying multi-locus chromatin contacts in human cells using tethered multiple 3C

Ferhat Ay, Thanh H. Vu, Michael J. Zeitz, Nelle Varoquaux, Jan E. Carette, Jean-Philippe Vert, Andrew R. Hoffman, William S. Noble

List of Figures

1 Number of restriction enzyme cut sites across the human genome. 2
2 Chromosome contact maps of different contacts types for KBM7-TM3C-1. 3
3 Ploidy track for select chromosomes from KBM7 TM3C data. 4
4 PCR verification of triples 1–10 listed in Main Figure 5. 5
5 Additional PCR experiments for triples 5 and 6. 6
6 Methylation status of the distal contact partners of *IGF2-H19* ICR for triple 1. 7
7 Methylation status of the distal contact partners of *IGF2-H19* ICR for triple 2. 8
8 Methylation status of the distal contact partners of *IGF2-H19* ICR for triples 3 and 4. 9
9 Gene expression measured by RNA-seq for the *IGF2-H19* locus. 10
10 Gene-poor chromosome 18 does not colocalize strongly with other small chromosomes that are gene-rich. 11

List of Tables

1 **Sequences of primers used for PCR verification.** 12

Supplementary Figures

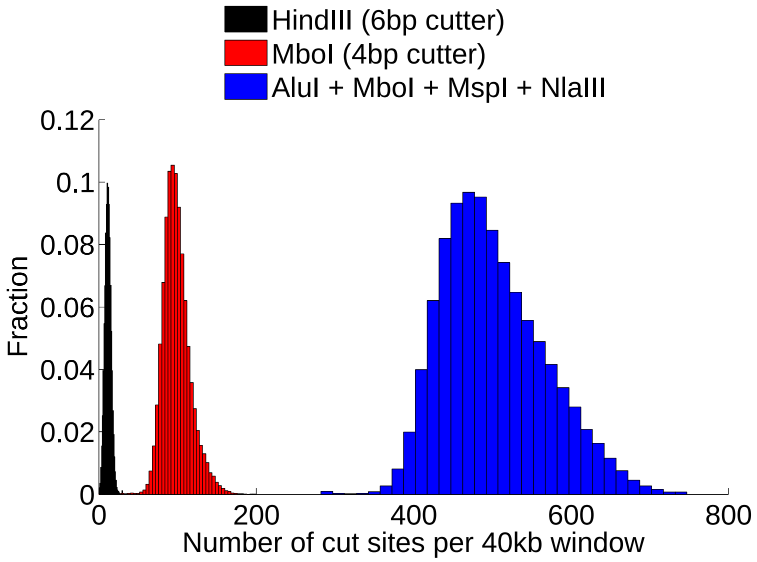

Supplementary Figure 1: Number of restriction enzyme cut sites across the human genome. Histograms of the number of cut sites within 40 kb windows across the human genome for each digestion system. HindIII, the most commonly used RE for creating Hi-C libraries, recognizes a 6 bp cut site, whereas AluI, MboI, MspI and NlaIII all cleave from 4 bp cut sites. For TM3C-1 libraries only MboI was used. For TM3C-4 libraries all four 4 bp cutters were used together to digest crosslinked chromatin. The theoretical resolution that can be achieved by each digestion system is inversely proportional to the RE cut site frequency. The mean number of cut sites per 40 kb suggests that TM3C-1 (99.5) and TM3C-4 (501.7) can achieve around 9 and 43 times higher resolution compared to using HindIII (11.7).

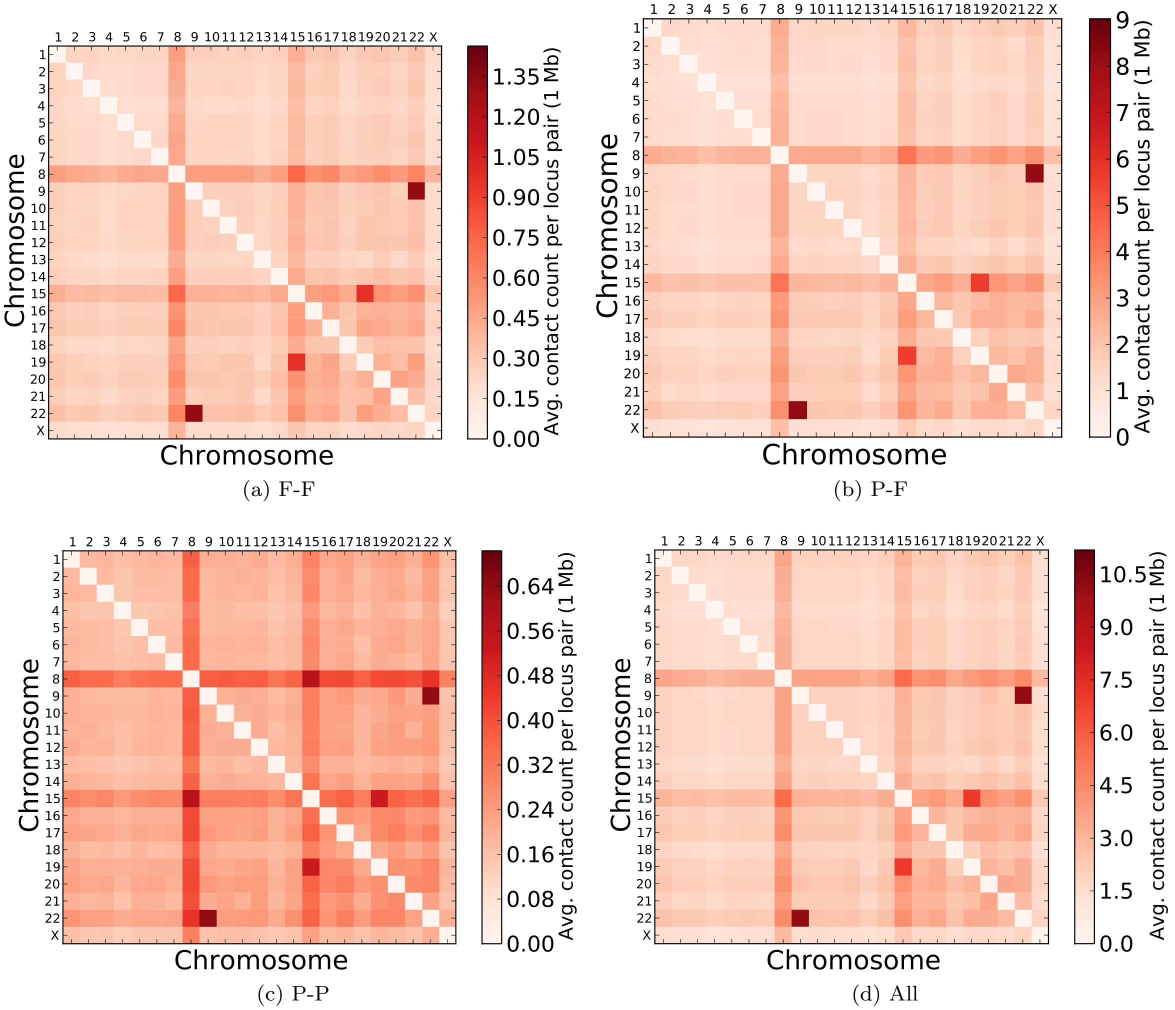

(a) F-F (b) P-F (c) P-P (d) All

Supplementary Figure 2: Chromosome contact maps of different contacts types for KBM7-TM3C-1. Pairwise raw contact counts are averaged over all pairs of mappable 1 Mb windows between the two chromosomes. Contacts that are of type (a) fully mapped/fully mapped (both ends mapped completely), (b) partially mapped/fully mapped (one end mapped completely and one end mapped after second phase of mapping) and (c) partially mapped/partially mapped (both ends mapped after second phase of mapping) are plotted separately. For (d) we aggregated all these three types of contacts.

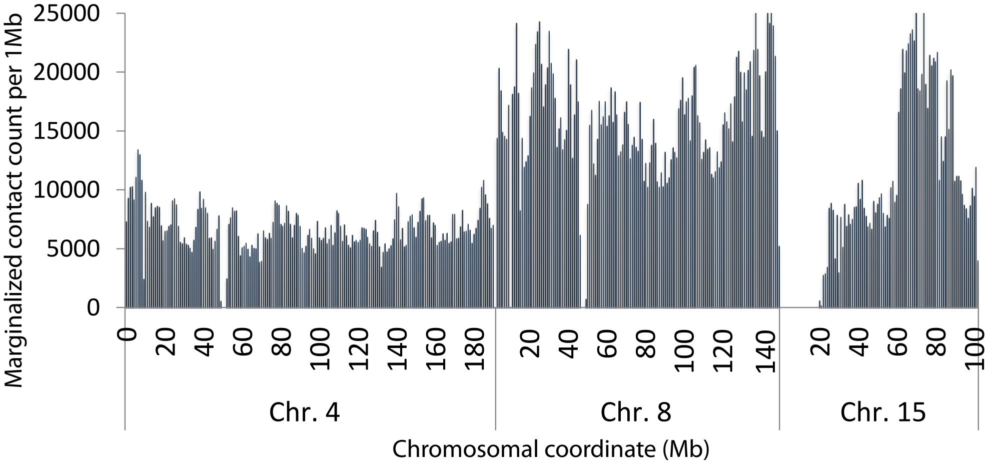

Supplementary Figure 3: Ploidy track for select chromosomes from KBM7 TM3C data. Plot of total contact count from each 1 Mb region to all other regions (both intra and interchromosomal) in the genome for a haploid (chr. 4), a diploid (chr. 8) and a partially diploid (chr. 15) chromosome. The diploid region of chromosome 15 is approximately 29 Mb (61–90 Mb).

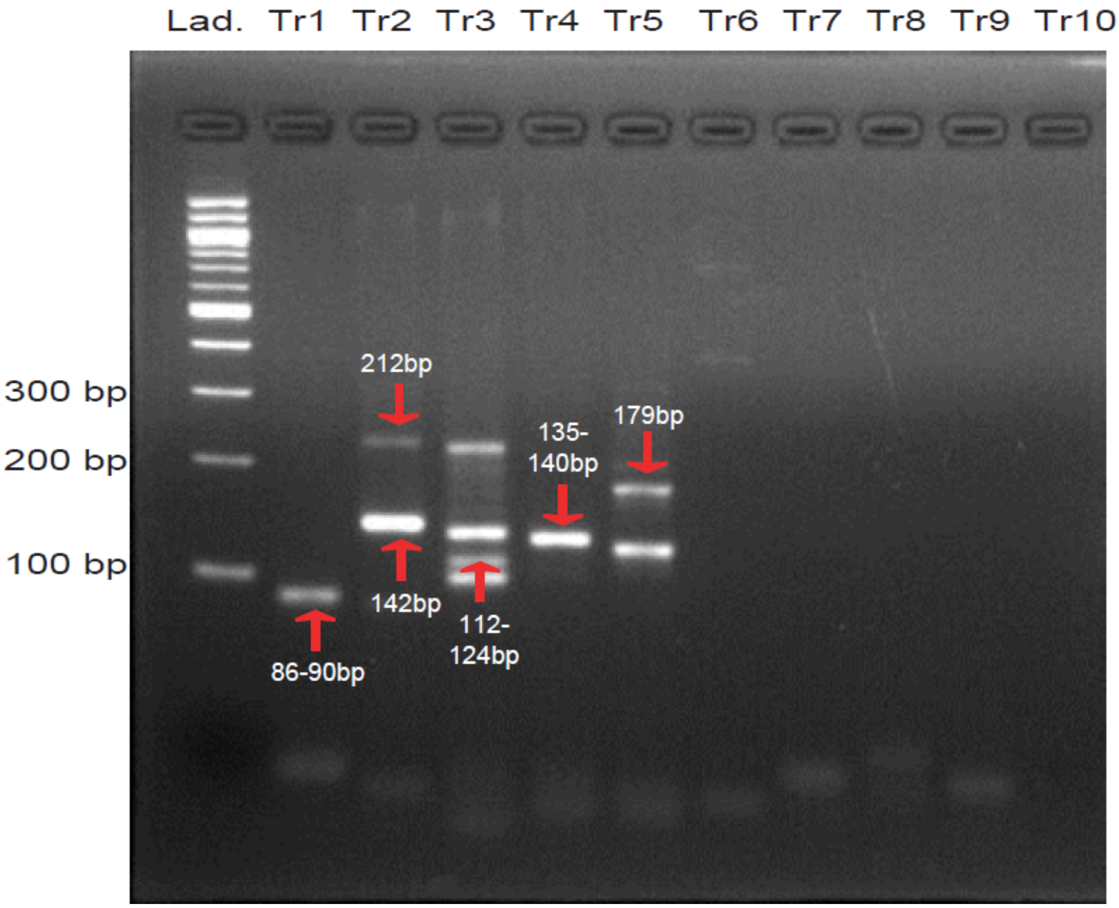

Supplementary Figure 4: PCR verification of triples 1–10 listed in Main Figure 5.
Lanes are: 100 bp ladder, triples 1 to 10. For each experiment all three primers (e.g., 1a+1b+1c) designed for that triple are used simultaneously. Expected product sizes for each triple are listed in Supplementary Table 1 and corresponding PCR products with approximate sizes are indicated by red arrows. Triples 1–5 showed PCR products in this gel, and triple 6 showed one product in another gel (Supplementary Fig. 5).

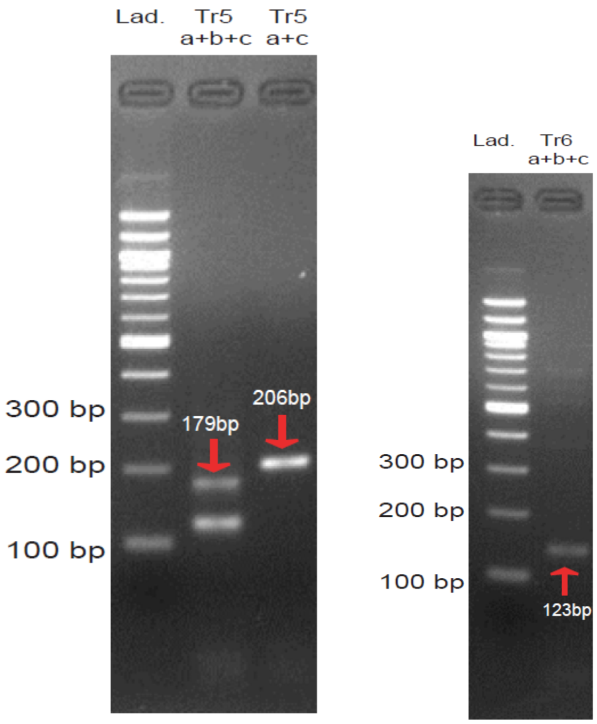

(a) Triple 5 only

(b) Triple 6 only

Supplementary Figure 5: Additional PCR experiments for triples 5 and 6.
(a) Triple 5 PCR gel. Lanes are: 100 bp ladder, 5a+5b+5c primers and only 5a+5c primers. (b) Triple 6 PCR gel. Lanes are: 100 bp ladder and 6a+6b+6c primers. Expected product sizes for each case are listed in Supplementary Table 1 and corresponding PCR products with approximate sizes are indicated by red arrows.

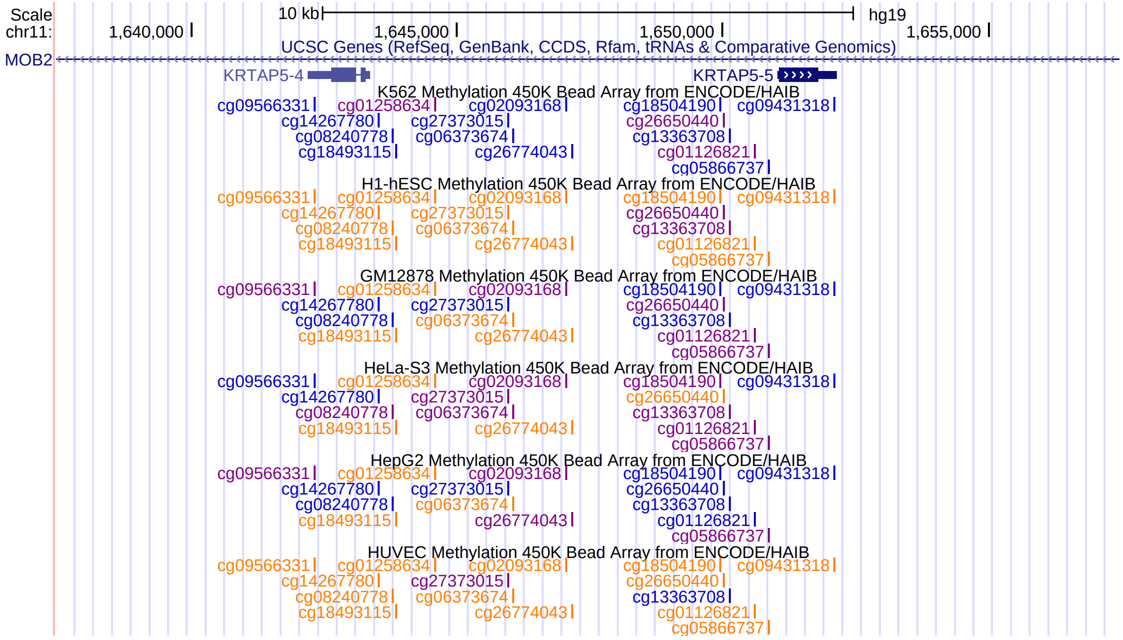

(a) Triple 1–end 1

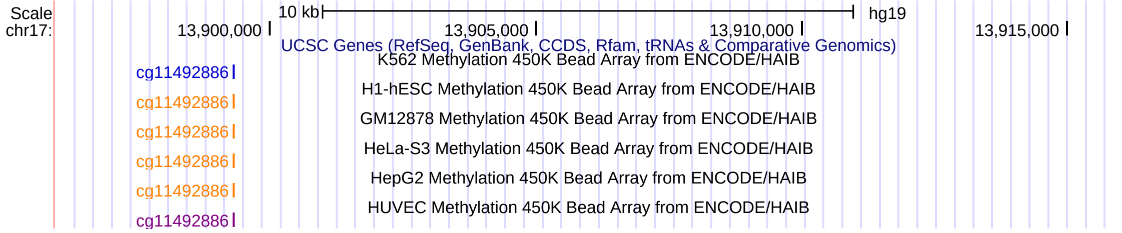

(b) Triple 1–end 3

Supplementary Figure 6: Methylation status of the distal contact partners of *IGF2-H19* ICR for triple 1.
UCSC genome browser snapshots of the tracks that display the methylation status of CpG dinucleotides in six ENCODE cell types for the two loci that are distal contact partners of ICR in triple 1. Methylation scores are color coded with orange for "methylated", purple for "partially methylated" and blue for "unmethylated". The figure displays a 20 kb region centered on (a) triple 1–end 1 located on chromosome 11, and (b) triple 1–end 3 located on chromosome 17. End 2 is located within 40 kb of the ICR.

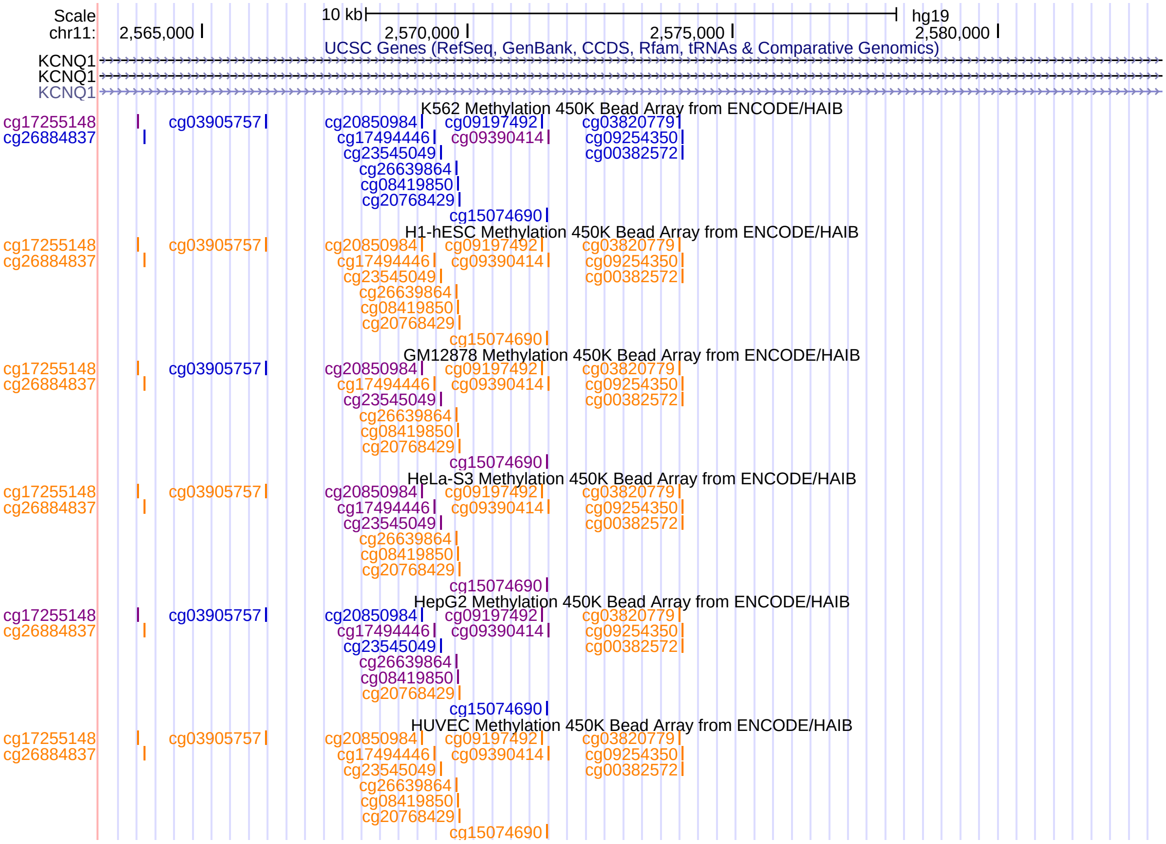

(a) Triple 2–end 2

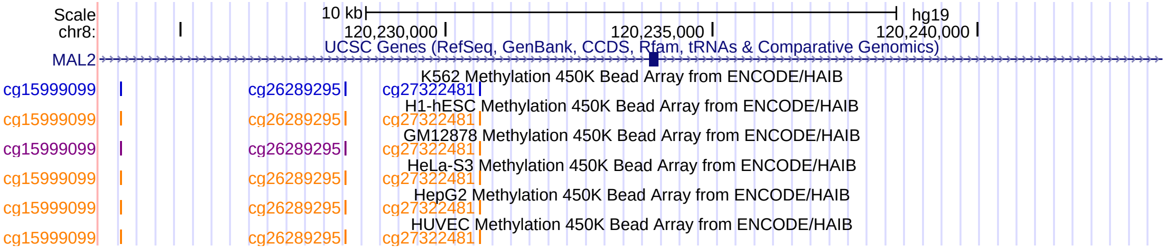

(b) Triple 2–end 3

Supplementary Figure 7: Methylation status of the distal contact partners of *IGF2-H19* ICR for triple 2.

Similar snapshots as Supplementary Fig. 6 above for 20 kb region centered on (a) triple 2–end 2 located on chromosome 11, and (b) triple 2–end 3 located on chromosome 8. End 1 is located within 40 kb of the ICR.

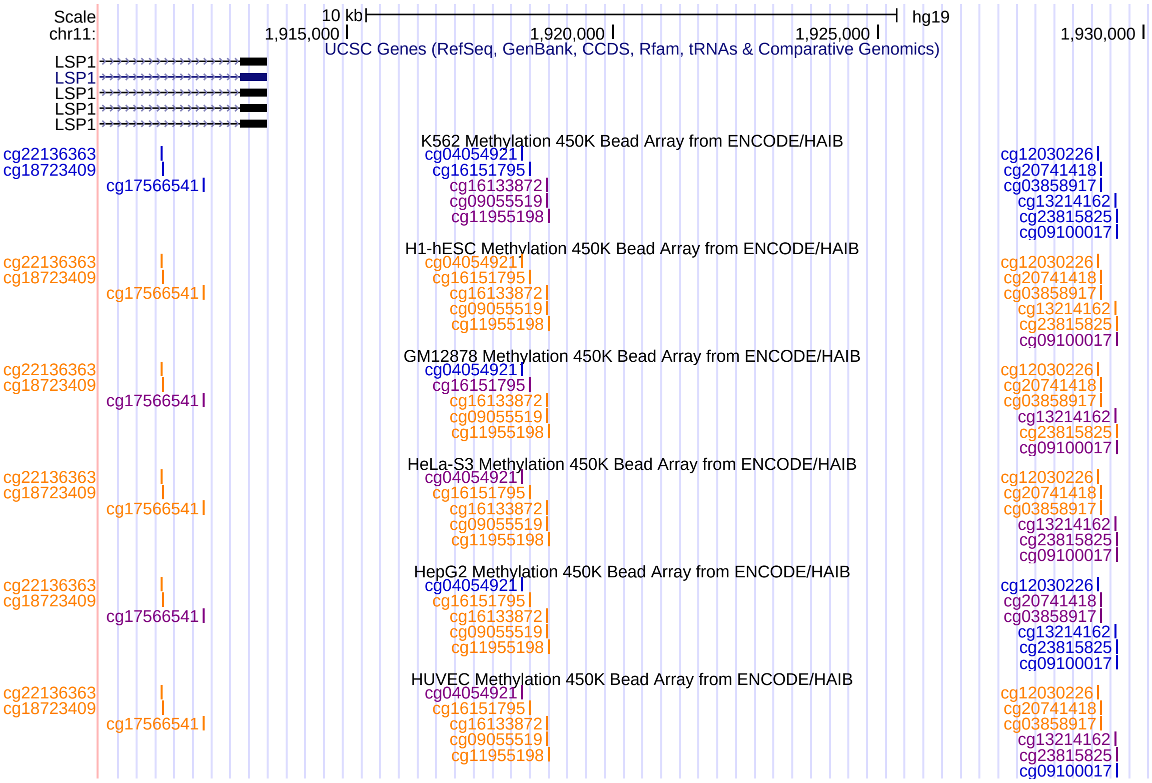

(a) Triple 3–end 1

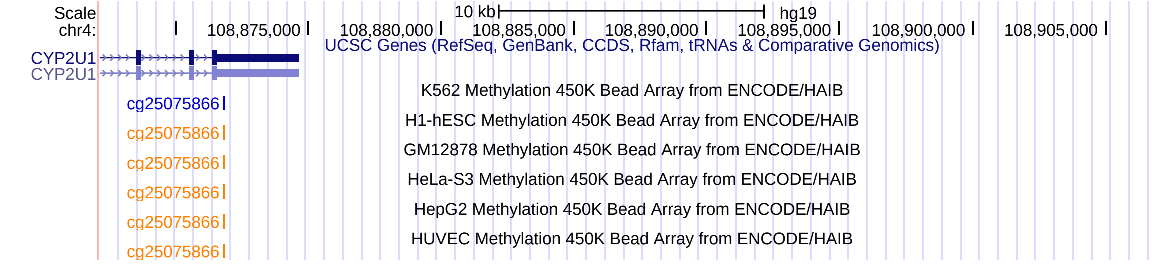

(b) Triple 4–end 3

Supplementary Figure 8: Methylation status of the distal contact partners of *IGF2-H19* ICR for triples 3 and 4.
Similar snapshots as Supplementary Fig. 6 above for (a) 20 kb region centered on triple 3–end 1 located on chromosome 11, and (b) 40 kb region centered on triple 4–end 3 located on chromosome 4. Ends 2 and 3 for triple 3, and ends 1 and 2 for triple 4 are located within 40 kb of the ICR.

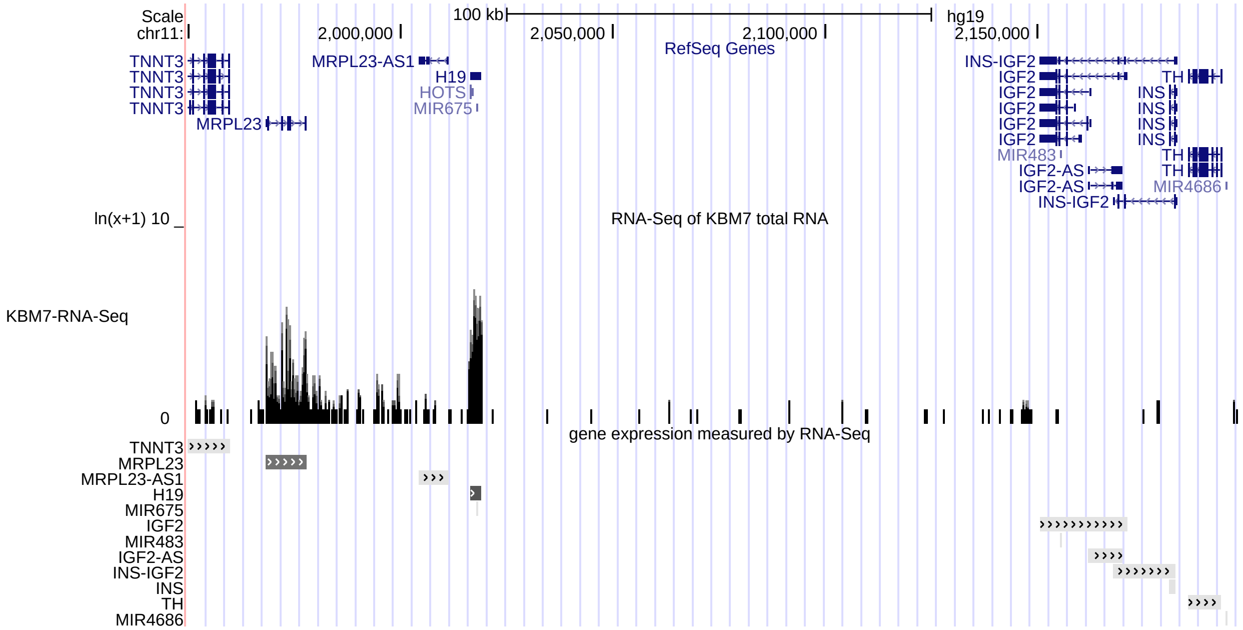

Supplementary Figure 9: Gene expression measured by RNA-seq for the *IGF2-H19* locus. Snapshot of 200 kb region taken from the KBM7 genome browser (Bürckstümmer et al.) that includes the *IGF2* and *H19* genes. RNA-seq measurements show that *H19* is expressed, whereas *IGF2* is not. This mode of *IGF2-H19* expression is consistent with the maternal expression pattern of human chromosome 11.

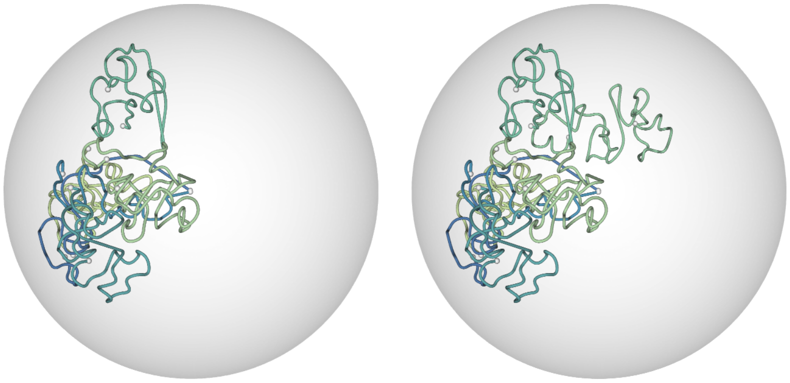

(a) Chromosomes 16, 17, 19, 20, 21, and 22

(b) Chromosomes 16–22

Supplementary Figure 10: Gene-poor chromosome 18 does not colocalize strongly with other small chromosomes that are gene-rich.

Supplementary Tables

Supplementary Table 1: **Sequences of primers used for PCR verification.**

These primers are designed to test 10 triple contacts listed in Main Figure 5 that involve *IGF2-H19* (ICR). Individual paired-end reads that produced each triplet are analyzed to construct forward/reverse primer pairs that are upstream/downstream of the MboI junction site that resulted in the corresponding chimera. All primer sequences are reported in 5′ to 3′ orientation even though reverse complements are used for reverse primers. The "Expected sizes" column lists the PCR products that are expected to be amplified when each primer triple is used.

Label	Chr.	Dist. to junction	Primer sequence	Strand	Expected sizes (bp)
1a	11	+44	5′-GTGACTGTGAACATTTTAACATGCATGTTTAACGC-3′	forward	
1b	11	-42	5′-TGCTGCACCCACATTAGCAGATTATCTCA-3′	reverse	86, 90
1c	17	-46	5′-AGGGTGATTTTCTTACTGTTTGTAAATAGTGCC-3′	reverse	
2a	8	+121	5′-AGGTGGTAGTCAGAGAATCAGTAAAG-3′	forward	
2b	11	+51	5′-TATAAGCCAAGGAGAGAGGCCTTGGAG-3′	forward	142, 212
2c	11	-91	5′-ACCCTTTCTCTTTTCCCCATTGGTGGTG-3′	reverse	
3a	11	-73	5′-TGTGAGCTGGTGCCAAGGACAGAGGCATCA-3′	reverse	
3b	11	-39	5′-CTCTTCCTTTTGGGGTGAAGACTGTCACCTTCTG-3′	reverse	112, 124
3c	11	+51	5′-ATTCAGAGGCATGACAGTCTCAAGTTCTTGGGA-3′	forward	
4a	11	-61	5′-TTGGCCACGGGCTCTGGAGGCCAGTGCCT-3′	reverse	
4b	4	+74	5′-GTAGGGAGGGAGAAACGGTAATGCTGGTCA-3′	forward	135, 140
4c	11	+79	5′-GGAGGCTCAGGTGAGCCCAGGTCTCCCTCTC-3′	forward	
5a	11	-131	5′-CACCATCCTCCCTCCTGAGAGCTCATTCACTCC-3′	reverse	
5b	11	+48	5′-GCAGCAGTGGCGCTCCCAGCTCTTTAGCA-3′	forward	179, 206
5c	11	+75	5′-TCGTAGGAGACTTTCACGGAGTGCCTGGTCTCC-3′	forward	
6a	2	+61	5′-GCTTATTCTCCATCGGTTTCTAAAGTTGTTCAT-3′	forward	
6b	11	-62	5′-ATTTCATCTCTGACCCAACCAATCAGCACTCCCTA-3′	reverse	96, 123
6c	4	+35	5′-ATTGTTTCCCAGTTCTGGAGTCCAGAAGTCCAA-3′	forward	
7a	11	+73	5′-TGCTTGCTCCTCCGGATGTCCCCTGTGTTTT-3′	forward	
7b	5	+88	5′-CCCAAAGTCATTGATATGGTTTGGCTGCATGTC-3′	forward	130, 145
7c	5	-57	5′-TGCTGATGAATATCTTGGCATCTAGGGGTCAAA-3′	reverse	
8a	5	+47	5′-CAGAAGTTAGGAGAGTCTTGAGTGTGCCTGTTT-3′	forward	
8b	11	+74	5′-TGTGGGCAAATTCACCTCTCCACGTGCCAACTA-3′	forward	154, 171
8c	15	-97	5′-AAAAATATGTTTCCCAGAAACTAGAGACTGGAG-3′	reverse	
9a	7	+61	5′-TGCCCATAGAAACAATTTACTCCAAGGGTCAAT-3′	forward	
9b	11	+49	5′-ACACCCGAGCCATCGAACATCCTAACCCCATCA-3′	forward	98, 110
9c	5	-37	5′-AGCACATGCTAATGCTATCATGAAGTCATACAC-3′	reverse	
10a	8	+55	5′-CCTCTTGTATTTGCTTTTTCCTCTTATCTCTCT-3′	forward	
10b	11	+49	5′-ACACCCGAGCCATCGAACATCCTAACCCCATCA-3′	forward	113, 119
10c	12	-64	5′-TCCCTCTCTCTTTTGTTTTTTGTACTTTATTTG-3′	reverse	
